# Supplementary material for: Association between oral microbiome and seven types of cancers in East Asian population: a two-sample Mendelian randomization analysis
Source: Front Mol Biosci. 2023 Nov 21;10:1327893. doi: 10.3389/fmolb.2023.1327893 (PMC10702768; doi:10.3389/fmolb.2023.1327893)
Supplement: Supplementary file 1 [file DataSheet1.ZIP › Supplementary Materials/MR plots of saliva/saliva═╝/ovarian cancer/pheno.429_to_ovarian cancer_forest.pdf]

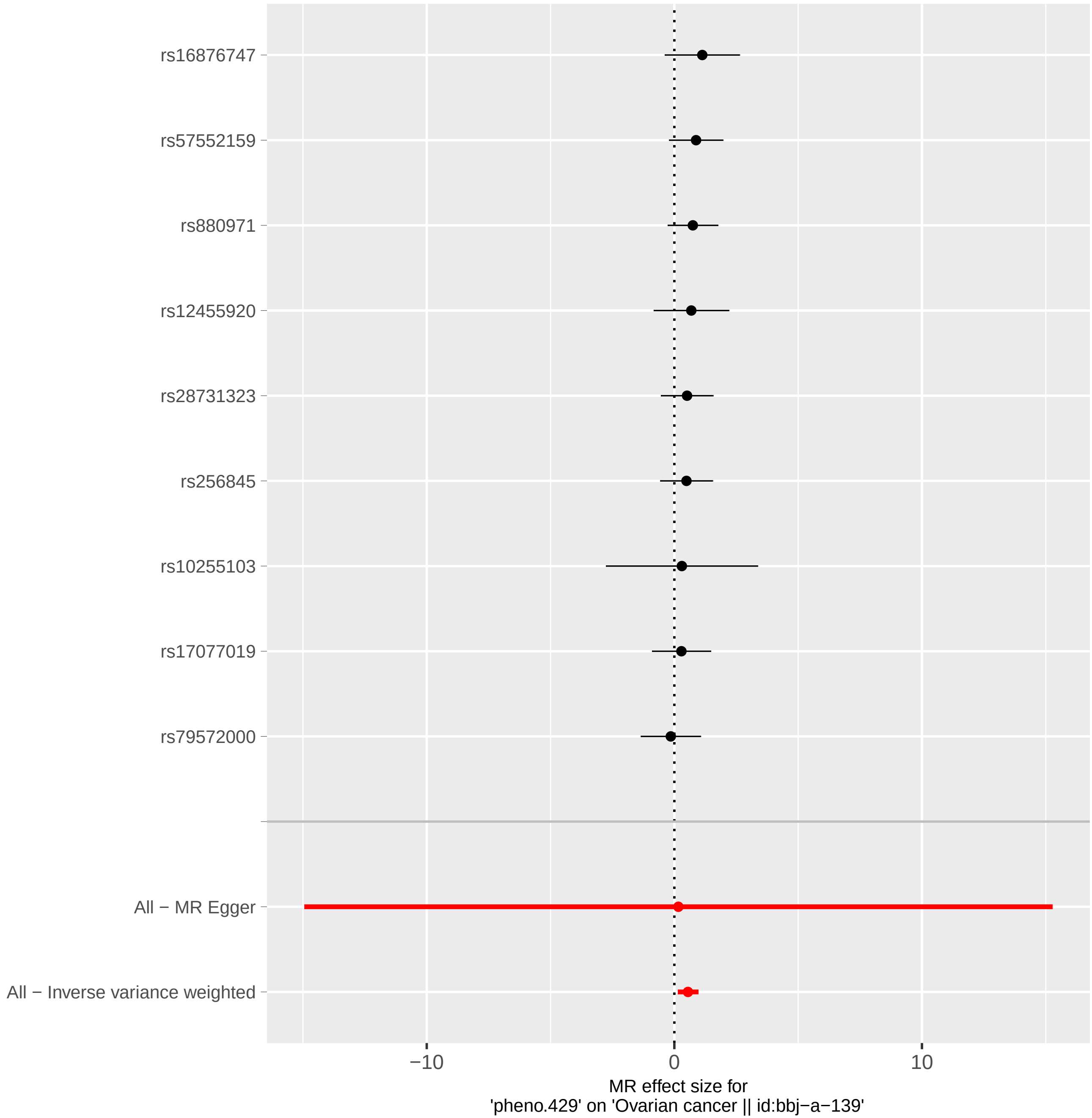

rs16876747
rs57552159
rs880971
rs12455920
rs28731323
rs256845
rs10255103
rs17077019
rs79572000
All − MR Egger
All − Inverse variance weighted
−10
0
10
MR effect size for
'pheno.429' on 'Ovarian cancer || id:bbj−a−139'
